# Clinical profiling of patients admitted with acute heart failure: a comprehensive survival analysis

**DOI:** 10.3389/fcvm.2024.1381514

**Published:** 2024-05-21

**Authors:** Raquel López-Vilella, Borja Guerrero Cervera, Víctor Donoso Trenado, Luis Martínez Dolz, Luis Almenar Bonet

**Affiliations:** ^1^ Heart Failure and Transplant Unit, Hospital Universitari i Politècnic La Fe, Valencia, Spain; ^2^ Cardiology Department, Hospital Universitari i Politècnic La Fe, Valencia, Spain; ^3^ Centro de Investigación Biomédica en Red de Enfermedades Cardiovasculares (CIBERCV), Instituto de Salud Carlos III, Madrid, Spain

**Keywords:** heart failure, clinical profiles, treatment, prognosis, survival

## Abstract

**Background:**

In heart failure (HF), not all episodes of decompensation are alike. The study aimed to characterize the clinical groups of decompensation and perform a survival analysis.

**Methods:**

A retrospective study was conducted on patients consecutively admitted for HF from 2018 to 2023. Patients who died during admission were excluded (final number 1,668). Four clinical types of HF were defined: low cardiac output (*n*:83), pulmonary congestion (*n*:1,044), mixed congestion (*n*:353), and systemic congestion (*n*:188).

**Results:**

The low output group showed a higher prevalence of reduced left ventricular ejection fraction (93%) and increased biventricular diameters (*p* < 0.01). The systemic congestion group exhibited a greater presence of tricuspid regurgitation with dilatation and right ventricular dysfunction (*p*:0.0001), worse renal function, and higher uric acid and CA125 levels (*p*:0.0001). Diuretics were more commonly used in the mixed and, especially, systemic congestion groups (*p*:0.0001). The probability of overall survival at 5 years was 49%, with higher survival in pulmonary congestion and lower in systemic congestion (*p*:0.002). Differences were also found in survival at 1 month and 1 year (*p*:0.0001).

**Conclusions:**

Mortality in acute HF is high. Four phenotypic profiles of decompensation differ clinically, with distinct characteristics and varying prognosis in the short, medium, and long term.

## Introduction

Heart failure (HF) can be considered a severe and chronic disease (1). However, far from remaining stable over time, it exhibits a clear tendency to progress towards clinical deterioration in affected patients (2). In this clinical evolution, the natural history of the disease shows that there are, with varying frequency, episodes of exacerbation and decompensation that necessitate hospitalization (3). Not all episodes of instability are the same, with different forms of decompensation ranging from scenarios of low cardiac output to clinical pictures of pulmonary or systemic congestion, or both (4). Each of these has a distinct clinical-analytical profile, requiring a different therapeutic approach and the adoption of a personalized prognostic evolutionary perspective (5, 6). Some of these clinical patterns have traditionally been associated with a worse prognosis (low output) (7). However, the clinical and analytical characterization, as well as the prescribed treatment at discharge, have not been clearly defined for all these profiles, revealing a lack of typification and analysis of the specificities of each. On the other hand, differences in survival among types of clinical decompensation have not been thoroughly investigated. The hypothesis when formulating this study was that since the clinical decompensation profile of HF patients admitted for HF is different, clinical characteristics, analytical alterations, prescribed treatment at discharge, and medium to long-term survival could also differ. Thus, the primary objective of the study was to analyze the clinical and analytical characteristics of patients admitted for heart failure according to the clinical decompensation profile. The secondary objective was to conduct a comparative analysis of overall survival and each group's long-term survival (5 years).

## Materials and methods

Retrospective longitudinal study based on patient data admitted to the Cardiology Department of a referral hospital. The study enrolled consecutive patients diagnosed with HF, including those with low cardiac output, pulmonary congestion, mixed congestion, and systemic congestion. The inclusion period spanned five years (June 2018–June 2023). Patients who died during hospitalization (*n*: 59) were excluded, as the primary goal was to understand the treatment that improved the patient's condition and enabled hospital discharge. The total number of patients included in the study was 1,668. The diagnosis was made following the criteria of the 2021 European Heart Failure Guidelines (8).

Variable definitions were established prior to the initiation of the database. (a) Low output: presence of hypotension (defined as a systolic blood pressure less than 90 mmHg) and hypoperfusion [defined as with peripheral coldness, oliguria (urine output <500 ml in 24 h), requiring inotropic support (dobutamine alone or with noradrenaline) for clinical stabilization]. (b) Pulmonary congestion: crackles in both lung fields and/or chest x-ray with pleural effusion and/or alveolar, interstitial, or mixed pattern without peripheral or minimal edema. Patients in whom congestion was confirmed by lung ultrasound also entered this group, although lung ultrasound was not a mandatory requirement for diagnosis. (c) Mixed congestion: pulmonary congestion defined as stated above plus significant edema in lower limbs (at least moderate degree) and/or abdomen with ascites. (d) Systemic congestion: Significant edema in lower limbs (at least moderate degree) and/or abdomen with ascites and/or hepatojugular reflux/hepatomegaly but without pulmonary congestion. Patients in whom congestion was confirmed by ultrasound (VExUS, venous excess ultrasound score) also entered this group, although VExUS was not performed routinely on all patients and this examination was not a mandatory requirement for diagnosis.

Ejection fraction was considered preserved when equal to or greater than 50% and reduced when below this threshold in the echocardiographic study conducted during the early days of hospital admission.

Medical history, echocardiographic parameters, admission laboratory results, and prescribed treatment at hospital discharge were analyzed and compared. Overall survival and survival within each subgroup were also compared during the study period (5 years).

Data entry into the database was completed on the day of hospital discharge. To minimize errors, data collection and entry were performed by personnel experienced in managing these patients, specifically by the same cardiologists belonging to the HF Unit.

The study received approval from the Biomedical Research Ethics Committee of the hospital, adhering to the ethical principles for medical research in human subjects as defined by the Declaration of Helsinki.

### Statistical analysis

Qualitative variables were expressed as numbers and percentages, and quantitative variables as median and interquartile range (non-normal distribution, *p* < 0.05 in the Kolmogorov–Smirnov test). Comparison between quantitative variables was performed using the Kruskal–Wallis ANOVA. For comparative analysis between qualitative variables, Pearson's *χ*^2^ test was applied. Survival curves were calculated using the Kaplan–Meier method, and comparisons were made using the Log Rank test. The multivariate analysis was conducted using Cox regression (Hazard Ratio), with the dependent variable being mortality and the independent variables being those considered of interest; the method employed was “Enter”. A *p*-value of <0.05 was considered significant. Statistical analysis utilized SPSS Statistics Version 27® software and Stata Statistics/Data Analysis 16.1, serial number 501606323439. Graphs were created using the SPSS program and modified with PowerPoint. The database was designed with Access and completed at the patient's discharge, excluding survival follow-up. Both programs are part of the Microsoft Office Professional Plus 2019 statistical package.

## Results

### Baseline characteristics

Some differences were found in the clinical profile among the study groups, particularly in the low output group, where younger males were more prevalent (*p*: 0.0001). Among the congestive groups, younger male patients were predominantly in the systemic congestion group (*p*: 0.0001). The most prevalent underlying heart condition in the low output group was ischemic heart disease, followed by idiopathic dilated cardiomyopathy. In the other groups, valvulopathies and ischemic heart disease were predominant (*p*: 0.0001). Relevant medical history (renal dysfunction and atrial fibrillation) was mostly present in the mixed and systemic congestion group (*p* < 0.01). These data can be observed in Table 1.

**Table 1:** Baseline characteristics by profile.

Study groups	Low output (83)	Pulmonary congestion (1,044)	Pulmonary + systemic congestion (353)	Systemic congestion (188)	*p*
Age (years)*	65 (15)	76 (16)	74 (19)	71 (17)	0.0001
Sex (males) (*n*, %)	66 (80)	577 (55)	214 (61)	125 (67)	0.0001
*Baseline cardiopathy* (*n*, %)					0.0001
Hypertensive	7 (8)	162 (16)	34 (10)	24 (13)	
iDCM	24 (29)	115 (11)	37 (11)	18 (10)	
Ischaemic	32 (39)	302 (29)	98 (28)	51 (27)	
Valvulopathy	10 (12)	305 (29)	100 (28)	52 (28)	
Others	10 (12)	160 (15)	84 (24)	43 (23)	
*Antecedents* (*n*, %)					
Previous CVS	10 (12)	213 (20)	80 (23)	53 (28)	0.069
Hypertension	54 (65)	846 (81)	276 (78)	141 (75)	0.01
Dyslipidaemia	47 (57)	653 (63)	202 (57)	105 (56)	0.256
Diabetes mellitus	38 (46)	463 (44)	165 (47)	71 (38)	0.520
COPD	10 (12)	155 (15)	71 (20)	20 (11)	0.068
Renal failure	21 (25)	320 (31)	183 (52)	84 (45)	0.0001
AF	38 (46)	585 (56)	224 (64)	123 (65)	0.009
CABG	2 (2)	75 (7)	28 (8)	14 (7)	0.376
De novo HF (*n*, %)	20 (24)	282 (27)	78 (22)	24 (13)	0.001

(*n*, %) number and percentage.

AF, atrial fibrillation; CABG, coronary artery bypass graft surgery; CVS, cardiovascular surgery; COPD, chronic obstructive pulmonary disease; iDCM, idiopathic dilated cardiomyopathy.

* Median and interquartile range.

### Echocardiographic and analytical parameters

Many differences were identified. The low output group was markedly different from the others, with a significant proportion of patients having reduced left ventricular ejection fraction [LVEF] (93%) and larger biventricular diameters (*p* < 0.01). Among the other three groups, the systemic congestion group showed a higher incidence of significant tricuspid regurgitation (TR) with dysfunction and dilation of the right ventricle [RV] (*p*: 0.0001). Regarding analytical parameters, the low output group exhibited the most significant clinical differences, particularly with a higher elevation of hepatic biomarkers, troponin, NT-ProBNP, and ferritin (*p* < 0.001). In the other groups, the most relevant clinical differences were a progressive increase in plasma levels of creatinine, uric acid, and CA 125 among the pulmonary congestion, mixed congestion, and systemic congestion groups (Table 2).

**Table 2:** Clinical, echocardiographic and analytical parameters by profile.

Study groups	Low output (83)	Pulmonary congestion (1,044)	Pulmonary + systemic congestión (353)	Systemic congestion (188)	*p*
Trigger of cardiac decompensation (*n*, %)					0.0001
Arrhythmias	17 (20)	213 (20)	71 (20)	18 (10)	
Hypertension	1 (1)	115 (11)	15 (4)	5 (3)	
Infectious	12	137 (13)	43 (12)	17 (9)	
Ischemic	12 (14)	63 (6)	9 (3)	1 (1)	
Disease progression	32 (14)	319 (31)	121 (34)	116 (62)	
Treatment abandonment	2 (4)	9 (1)	7 (2)	2 (1)	
Unknown	6 (7)	103 (10)	44 (12)	10 (5)	
Others	1 (1)	85 (8)	43 (12)	19 (10)	
SBP (mmHg)*	109 (21)	135 (38)	133 (38)	122 (37)	0.0001
DBP (mmHg)*	72 (13)	77 (20)	71 (22)	73 (17)	0.0001
Reduced LVEF (*n*, %)	77 (93)	574 (55)	184 (52)	111 (59)	0.0001
LVEF (%)*	22 (22)	32 (22)	30 (36)	43 (22	0.0001
LVEDD*	60 (15)	52 (14)	52 (14)	52 (11)	0.005
LVESD*	52 (19)	38 (20)	38 (20)	38 (14)	0.0001
Mitral regurgitation (*n*, %)^#^	36 (43)	175 (17)	78 (22)	34 (18)	0.0001
Tricuspid regurgitation (*n*, %)^#^	29 (35)	146 (14)	85 (24)	68 (36)	0.0001
Mild tricuspid regurgitation (*n*, %)	15 (18)	161 (15)	42 (12)	33 (18)	0.0001
Moderate tricuspid regurgitation (*n*, %)	16 (19)	83 (8)	39 (11)	22 (12)	0.003
Severe tricuspid regurgitation (*n*, %)	13 (16)	63 (6)	46 (13)	46 (25)	0.0001
Reduced RVEF (*n*, %)	18 (22)	63 (6)	35 (10)	34 (18)	0.0001
RV dilatation (*n*, %)	43 (52)	198 (19)	127 (36)	88 (47)	0.0001
TAPSE (mm)*	16 (2)	16 (6)	13 (4)	15 (5)	0.002
S’(cm/s)*	9,4 (1.4)	8.6 (3.7)	7.3 (4.1)	8.7 (3.5)	0.029
PAPs (mmHg)*	50 (20)	51 (20)	47 (15)	55 (10)	0.052
Urea (mg/dl)*	58 (46)	49 (36)	60 (51)	88 (102)	0.0001
Creatinine (mg/dl)*	1.3 (0.7)	1.2 (0.7)	1.3 (1.0)	1.7 (1.7)	0.0001
GFR (ml/min/1.73 m^2^)*	56 (37)	58 (41)	48 (41)	37 (48)	0.0001
GOT/AST (U/L)*	65 (202)	23 (15)	22 (18)	28 (15)	0.0001
GPT/ALT (U/L)*	42 (96)	21 (19)	19 (16)	18 (69)	0.0001
usTnT (ng/L)*	145 (728)	38 (49)	54 (63)	57 (46)	0.0001
NT ProBNP (pg/ml)*	8,444 (11,460)	5,294 (8,158)	6,203 (9,391)	4,798 (7,696)	0.001
Sodium (mEq/L)*	137 (7)	140 (5)	139 (6)	138 (6)	0.0001
Potassium (mEq/L)*	4.2 (0.8)	4.3 (0.7)	4.4 (0.9)	4.4 (0.9)	0.947
Hemoglobin (g/dl)*	13.4 (2.6)	12.4 (2.7)	12.1 (3.3)	12.0 (3.2)	0.0001
Hematocrit (%)*	41 (7)	38 (8)	38 (9)	36 (9)	0.0001
Uric acid (mg/dl)*	7.7 (4.2)	7.8 (3.0)	8.5 (3.3)	9.2 (3.5)	0.0001
TSAT (%)*	17 (12)	17 (11)	17 (12)	18 (13)	0.898
Ferritin (ng/ml)*	240 (260)	158 (228)	145 (218)	157 (333)	0.0001
Hb1AC (%)*	5.9 (1.1)	6.0 (1.0)	6.1 (1.1)	5.9 (1.1)	0.003
CA125 (U/ml)*	135 (254)	57 (108)	113 (163)	149 (306)	0.0001

(*n*, %) number and percentage.

ALT (GPT), alanine aminotransferase; AST (GOT), aspartate aminotransferase; CA125, 125; Carcinoembryonic antigen GFR:DBP, diastolic blood pressure; glomerular filtration rate; Hb1AC, Glycated hemoglobin; TSAT, transferrin saturation; LVEDD, left ventricle end-diastolic diameter; LVEF, left ventricle ejection fraction; LVESD, left ventricule end-systolic diameter; NT-ProBNP, N terminal pro B type natriuretic peptide; PAPs, pulmonary artery systolic pressure; RV, right ventricle; RVEF, right ventricle ejection fraction; S’, tricuspid annular systolic velocity; SBP, systolic blood pressure; TAPSE, tricuspid annular plane systolic excursion; usTNT, ultrasensitive troponin T.

^#^ Moderate-severe to severe mitral and tricuspid regurgitation.

* Median and interquartile range.

### Discharge treatment

The most apparent differences included a higher use of diuretics in the mixed and, especially, systemic congestion groups (*p*: 0.0001). Detailed comparative analysis can be observed in Table 3.

**Table 3:** Discharge treatment by profile.

Study groups	Low output (83)	Pulmonary congestion (1,044)	Pulmonary + systemic congestion (353)	Systemic congestion (188)	*p*
IECA/ARB (*n*, %)	20 (24)	407 (39)	148 (42)	43 (23)	0.0001
ARNI (*n*, %)	36 (43)	219 (21)	81 (23)	30 (16)	0.0001
Bb (*n*, %)	65 (78)	762 (73)	258 (73)	117 (62)	0.001
MRA (*n*, %)	60 (72)	438 (42)	205 (58)	132 (70)	0.0001
SGLT2i (*n*, %)	42 (51)	365 (35)	138 (39)	79 (42)	0.0001
Loop diuretics (*n*, %)	71 (86)	981 (94)	335 (95)	186 (99)	0.0001
Thiazide (*n*, %)	13 (15)	157 (15)	95 (27)	66 (35)	0.0001
Tolvaptan (*n*, %)	11 (13)	31 (3)	2 (5)	34 (18)	0.0001
Acetazolamide (*n*, %)	2 (2)	10 (1)	11 (3)	13 (7)	0.0001

(*n*, %) number and percentage.

Bb, beta-blockers; IECA/ARB, angiotensin converting enzyme inhibitor/angiotensin II receptor antagonist; ARNI, neprilysin and angiotensin receptor inhibitor; MRA, mineralocorticoid receptor antagonist; SGLT2i, sodium-glucose cotransporter dual inhibitors.

### Survival analysis

**Figure 1:**
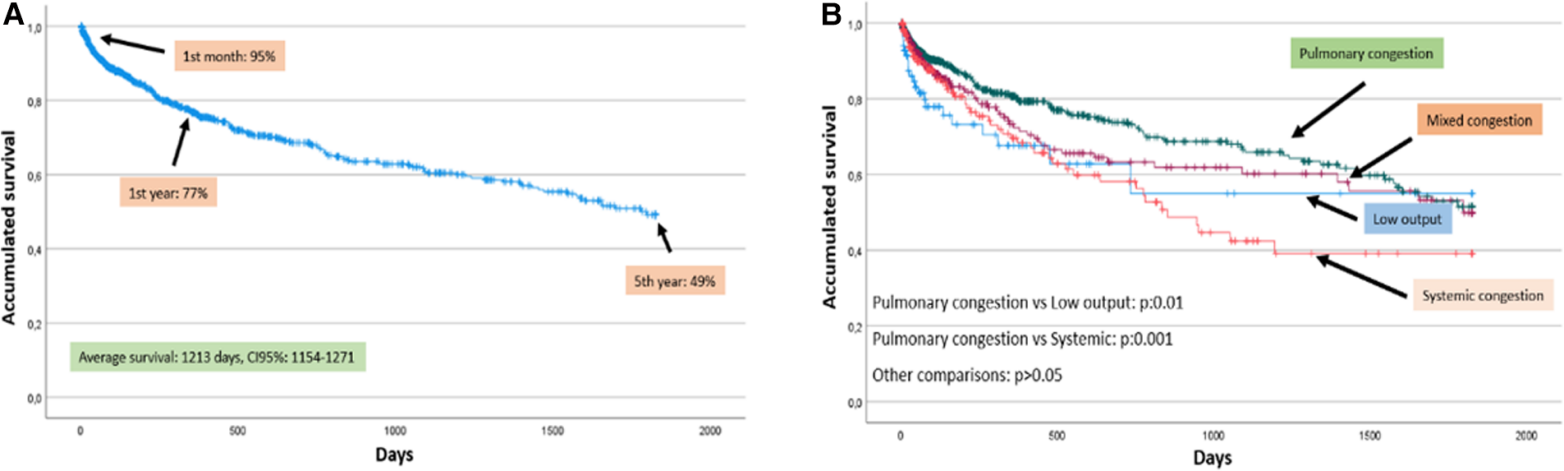
Overall and study group survival function. (**A**) Probability of survival for the entire series. (**B**) Probability of survival by study groups. *Median survival:* Low output group: 1,113 days, 95% CI: 845–1,382. Pulmonary congestion group: 1,288 days, 95% CI: 1,214–1,362. Mixed congestion group: 1,191 days, 95% CI: 1,064–1,317. Systemic congestion group: 990 days, 95% CI: 832–1,148. CI, confidence interval.

**Figure 2:**
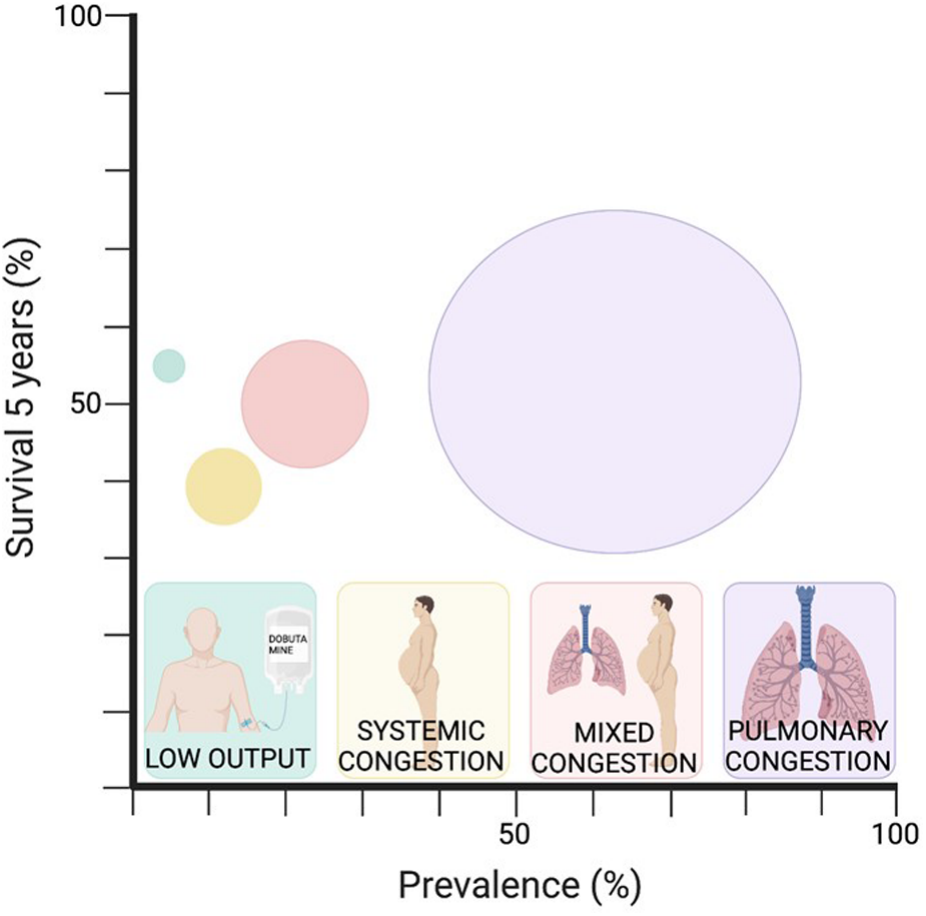
Circles size is proportional to the prevalence of each profile.

**Figure 3:**
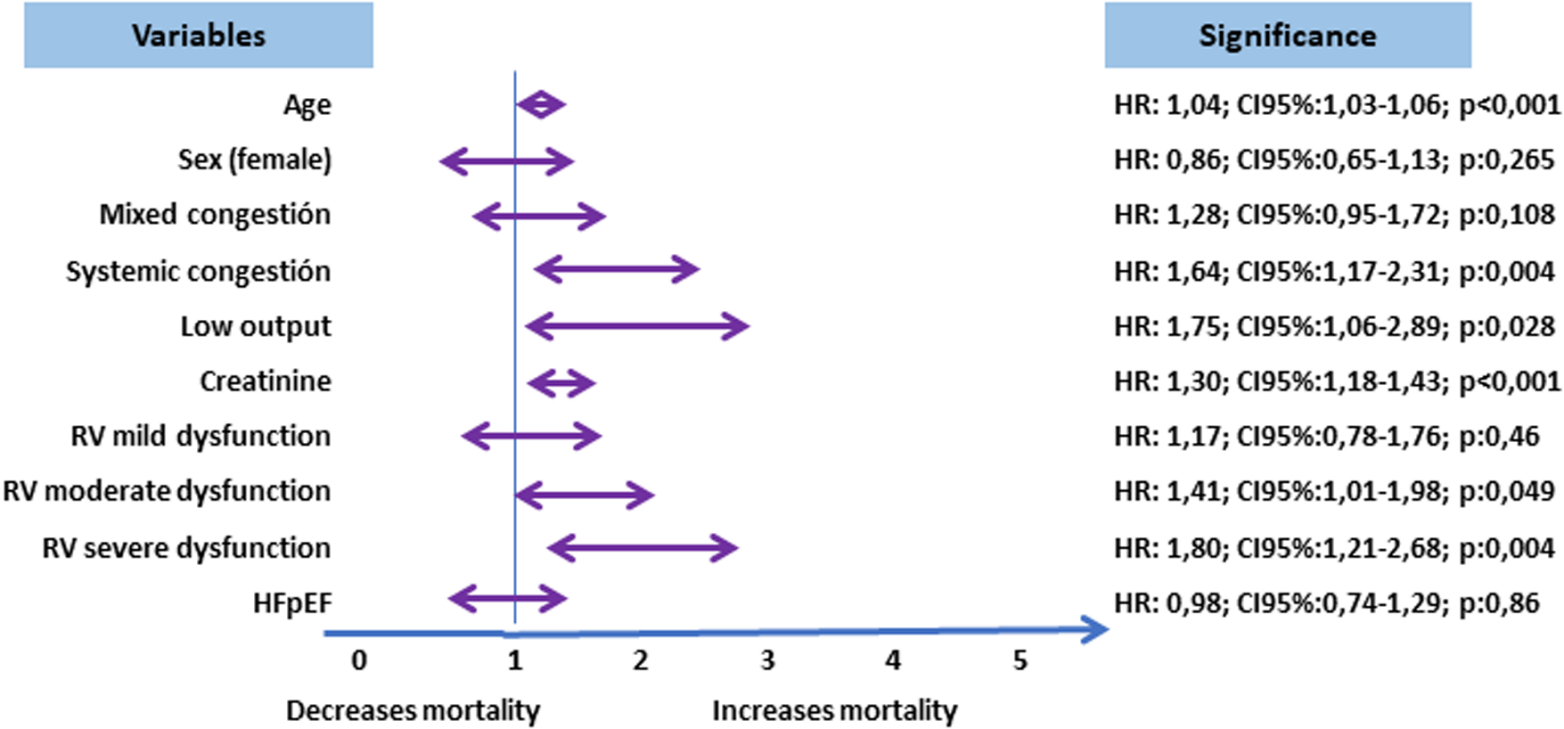
Multivariate analysis. Cox regression (Hazard Ratio). The congestion profiles are analyzed with respect to the pattern of pulmonary congestion. Right ventricular dysfunction is analyzed in relation to normal right ventricular function.

Mortality during the entire follow-up exceeded 50% at 5 years, with a gradual decrease from admission due to decompensation and an average survival of 1,213 days (Figure 1A). When comparing survival function by groups, it was evident that they were not all equal. Thus, the best survival was observed in the pulmonary congestion group and the lowest in the systemic congestion group, even below that of the low output group. The differences found were between pulmonary congestion vs. low output (*p*: 0.01) and vs. systemic congestion (*p*: 0.001) (Figure 1B). The comparison at different periods (1 month, 1 year, and 5 years) of the probability of survival by groups was different (*p* < 0.05), with lower survival at one month and one year in the low output group. There were also differences in the percentage of deceased individuals in each group (*p*: 0.0001) (Table 4 and Figure 2). In the multivariate analysis, age, systemic congestion, low cardiac output, renal dysfunction, and right ventricular dysfunction were found to be independent predictors of mortality (Figure 3).

**Table 4:** Probability of survival and number of exitus during follow-up.

Study groups	Low output (83)	Pulmonary congestion (1,044)	Pulmonary and systemic congestion (353)	Systemic congestion (188)	*p*
1 month^#^	86%	95%	95%	94%	0.004
1 year^#^	68%	81%	72%	70%	0.0001
5 years^#^	55%	52%	50%	39%	0.008
Exitus follow-up (*n*, %)	37 (45)	501 (48)	176 (50)	115 (61)	0.0001

(*n*, %) number and percentage.

^#^ Probability of survival.

## Discussion

Admissions due to decompensation in HF reduce patients' quality of life and shorten their survival probability (1–3, 7). However, the clinical picture of HF decompensation does not have a unique phenotypic profile; it can vary, leading to different clinical characteristics and prognoses (4). Currently, it is essential, for personalized precision medicine, to identify clinical phenotypes within the heart failure framework that help us correctly identify patients admitted for decompensation. This aids in conducting a functional and prognostic assessment, allowing for the establishment of individualized therapeutic and follow-up goals by identifying the risk related to the decompensation reason and, consequently, the evolutionary prognosis. This study aimed to identify the four clinical profiles that patients present upon admission for decompensations to understand their overall clinical characteristics and the probability of survival over a 5-year period.

It has been confirmed that there are four different clinical profiles into which all patients can be categorized. These profiles differ in terms of clinical characteristics, analytical parameters, echocardiographic studies, and differences in the prescription of cardioactive drugs at discharge. Additionally, there are differences in survival, with the highest in pulmonary congestion and the lowest in systemic congestion. In this study, we selected the four profiles that we believe encompass all clinical presentations of heart failure decompensation. These profiles are easily identifiable, both through analytical parameters (NT-ProBNP, CA 125) and clinical indicators (6), in a more objective manner than the classic determination of wet/dry or cold/hot. This classic determination is much more variable over time and examiner-dependent (9, 10). It also allows the exclusion of ejection fraction from the classification, which, in many cases, does not correspond to the clinical presentation. Additionally, we incorporated the mixed congestion pattern (systemic and pulmonary), not explicitly reflected in guidelines (8) or in most studies in this field (5, 11–13). However, in our study, it has a prevalence of 21.1% and presents characteristics regarding survival, evolution, and clinical features distinct from other congestion patterns and the low-output pattern.

It is important to note that in other studies, clinical profiles are classified as cold or hot based on perfusion status and as wet or dry based on congestion status (10, 13, 14). In this series, the low-output group includes patients who are also congestive (cold and wet). This distinction is crucial since the medical management of these patients classified in the same group will be different and includes the use of diuretics.

Among the baseline characteristics, differences exist between the groups, as reflected in other large studies on clinical profiles such as the EHFS II study [3,580 patients] (15) or the ESC-EORP-HFA Registry [7,865 patients] (16). In these studies, the average age of patients was around 70 years, with a male predominance (higher prevalence in the low-output group), coinciding with the findings of the present study. The most prevalent underlying heart condition in the low-output group was ischemic heart disease in both studies, whereas in the other groups, valvular heart disease was more prevalent in EHFS II, and ischemic heart disease was also prevalent in ESC-EORP-HFA. In ESC-EORP-HFA, renal dysfunction and atrial fibrillation were predominant in congestive patients, aligning with the results of this analysis.

Regarding echocardiographic characteristics, in the European ESC-HF-LT registry with 6,629 patients, the cardiogenic shock group showed the highest percentage of patients with reduced left ventricular ejection fraction (LVEF) and severe mitral regurgitation, similar to these results. Additionally, a certain percentage of patients (14.7%) presented with preserved LVEF. This finding can be justified by concurrent valvular diseases (mitral regurgitation and/or aortic stenosis), situations of hypovolemia (third space), diastolic dysfunction, or severe right ventricular dysfunction (10, 13, 17). Right ventricular dysfunction is more prevalent in low output and systemic congestion groups. This is due to the determining role that the RV plays physiopathologically in both conditions. Right ventricular dysfunction begins with initial myocardial injury, with the most common cause being left HF. The mechanism that causes right-sided failure initiates initial dyssynchrony, dilation, increased wall tension, and oxygen consumption, leading to a decrease in right ventricular contractile function. The interventricular septum shifts leftward due to ventricular interdependence, reducing left ventricular preload and afterload, leading to decreased cardiac output. The decrease in cardiac output limits coronary flow, which exacerbates right ventricular dysfunction, whose dysfunction causes systemic congestion due to increased central venous pressures; this causes more organ damage than the decrease in cardiac output alone. In this sense, right ventricular dysfunction has been associated with worse prognosis, consistent with the results of the present study. In acute HF, right dysfunction is associated with a higher risk of recurrent admissions (18), especially in HF with preserved ejection fraction (18, 19). No differences were found in our study regarding estimated pulmonary systolic pressure by echocardiography, although previous studies had reported a relationship between higher pulmonary systolic pressure values and poor prognosis in acute HF (19, 20). Finally, in the echocardiographic characteristics of the 4 study groups, it can be observed how severe TR is more frequent in the systemic congestion group, which is consistent with worse prognosis in this group, with TR being a known factor associated with poor prognosis and worse survival in acute HF, regardless of the presence of pulmonary hypertension (21). In relation to other types of ultrasound, it should be noted that in this study, ultrasound was not systematically conducted on patients to assess the degree of congestion. Although ultrasound is not typically employed routinely in the context of acute heart failure, it is undeniably an increasingly utilized tool. It is recognized that clinical and ultrasound indicators of congestion are highly prevalent in patients with acute heart failure, and that their combined assessment enhances risk stratification (22–24).

In reference to analytical values, it is consistent with the pathophysiology of each profile that higher levels of CA 125 and worse renal function are found in the systemic congestion group (6). According to current scientific literature, levels of the carbohydrate antigen CA 125 have been widely associated with the state of congestion, increasing with its severity (25). Furthermore, the elevation of this molecule has been linked to higher rates of readmission and adverse clinical outcomes (26, 27). Unfortunately, major series analyzing patients based on their HF profile do not include this value in their results. This study represents the first European series to analyze this parameter within the specific clinical profiles of HF selected for this research (10, 13–15). Notably, the results highlight a progression within the congestive groups, where renal function deteriorates progressively, and CA 125 increases from the pulmonary congestion group, through mixed congestion, and finally to systemic congestion. These findings align with the pathophysiology of CA-125 and its association with clinical progression and survival, as demonstrated in the results (28).

Regardless of the analyzed HF profile, the mortality throughout the series was high, exceeding 50% at 5 years, with a gradual decrease from the decompensation admission and a mean survival of 1,213 days. These figures align with other large studies. The ECHOES study, involving 3,960 patients with a 5-year follow-up, reported survival rates around 50% (29). A Spanish study showed mortality values exceeding 40% at 5 years (30). Some studies even indicate mortality rates exceeding 70% at 5 years, regardless of the type of HF presented by patients (31, 32).

When analyzing survival by groups, the group with the lowest survival within the first month and the first year is the low output group. However, at a 5-year follow-up, the profile offering better survival is the pulmonary congestion group, while the systemic congestion group exhibits lower survival (with a median nearly 300 days less than the former), even with worse figures than the low output group. In the multivariate analysis, both systemic congestion and low cardiac output profile turned out to be independent predictors of mortality compared to the lower mortality profile (pulmonary congestion). It can be observed that the low output group experiences a rapid decline in survival initially but then stabilizes, whereas the systemic congestion group's curve shows a more gradual decline without stabilizing. In fact, its median survival is 123 days less than the low output group. In most reviewed series, the low output group initially presents higher mortality figures. However, when studying data beyond the first year, survival curves start to converge, and other clinical profiles equal or surpass the low output group, such as the systemic congestion group (28, 30). This may be justified by the fact that although the low output profile with systemic hypoperfusion has higher short-term mortality initially (32), follow-up has described congestion at discharge, renal dysfunction, and elevated proBNP and CA-125 levels as mortality predictors in HF patients (9, 23, 27). All of these factors are more prevalent in the systemic congestion group, which showed the worst 5-year survival.

The study's potential limitations are associated with inherent biases in retrospective studies. Routine ultrasound techniques were not used to classify patients in each group, although signs, symptoms and other complementary examinations were used. Drug prescriptions upon discharge depended on the attending physician during admission, introducing possible variability in medical criteria. However, clinical practice guidelines exist in the cardiology department, established by the HF Unit and utilized by all specialists treating HF patients. It was also not possible to assess whether the lack of prescription was due to side effects or additional comorbidities, introducing a potential measurement bias. Residual congestion at hospital discharge has also not been evaluated. Finally, patients who died during hospitalization were excluded, and this can be understood as a possible limitation. Nevertheless, the study involved a significant number of patients included over 5 consecutive years with predefined variables at the initial inclusion and in a single center, ensuring homogeneity in the assignment to a specific clinical group. Additionally, having hospitalized patients allowed for on-site necessary examinations to precisely define the decompensation pattern, avoiding selection biases. Both data collection from electronic records and input into the computer program were carried out by cardiologists from the HF Unit, enhancing data reliability by reducing methodological errors in transcription and interpretation, thereby avoiding information biases.

## Conclusion

There are four clinical profiles among patients admitted for decompensated HF: low cardiac output, isolated pulmonary or systemic congestion, and mixed congestion. The differences between them are marked by clinical characteristics, analytical parameters, echocardiographic studies, and variations in the prescription of cardioactive drugs at discharge. There are significant differences in long-term survival between the profiles, such that cases of pulmonary congestion have a better prognosis, while cases of systemic congestion result in more fatalities during follow-up.

## Data Availability

The raw data supporting the conclusions of this article will be made available by the authors, without undue reservation.
